# Modulation of a rapid neurotransmitter receptor-ion channel by membrane lipids

**DOI:** 10.3389/fcell.2023.1328875

**Published:** 2024-01-11

**Authors:** Francisco J. Barrantes

**Affiliations:** Biomedical Research Institute (BIOMED), Catholic University of Argentina (UCA)–National Scientific and Technical Research Council (CONICET), Buenos Aires, Argentina

**Keywords:** plasma membrane, plasmalemma, membrane lipids, lipid influence on membrane proteins, cholesterol, nicotinic acetylcholine receptor

## Abstract

Membrane lipids modulate the proteins embedded in the bilayer matrix by two non-exclusive mechanisms: direct or indirect. The latter comprise those effects mediated by the physicochemical state of the membrane bilayer, whereas direct modulation entails the more specific regulatory effects transduced via recognition sites on the target membrane protein. The nicotinic acetylcholine receptor (nAChR), the paradigm member of the pentameric ligand-gated ion channel (pLGIC) superfamily of rapid neurotransmitter receptors, is modulated by both mechanisms. Reciprocally, the nAChR protein exerts influence on its surrounding interstitial lipids. Folding, conformational equilibria, ligand binding, ion permeation, topography, and diffusion of the nAChR are modulated by membrane lipids. The knowledge gained from biophysical studies of this prototypic membrane protein can be applied to other neurotransmitter receptors and most other integral membrane proteins.

## 1 Introduction

Chemical signaling via hormones or neurotransmitters at the plasmalemma was acquired early in phylogeny. Though electrical signaling via low resistance intercellular gap junctions and extracellular electrical fields generated by the electrical activity of neurons are also operative mechanisms (Faber and Pereda, 2018), chemical signaling provided specificity to the messages conveyed by the coding molecules. Such an evolutionary event prompted the emergence of cell-surface receptors approximately 4,000 million years ago. One such group of membrane-associated receptors is the pentameric-gated ion channel (pLGIC) superfamily initially in prokaryotes and subsequently in eukaryotes (Ortells et al., 1992; Barrantes, 2015). The pLGIC superfamily includes the following neurotransmitter receptor families: the nicotinic acetylcholine receptors (nAChRs), the γ-aminobutyric acid (type A and C) receptors, the glycine receptors, subtype 3 of the serotonin receptor family, and the glutamate-gated chloride channel (Zoli et al., 2018). pLGICs are built following a common architecture: five subunits in a pseudo-symmetric layout surrounding an ion-permeation pathway, the ionic pore. The structural similitude is related to the evolutionary history and conservation of these membrane-bound proteins (Stroud and Finer-Moore, 1985; Le Novère and Changeux, 1995; Ortells, 1998; Barrantes, 2015; Tessier et al., 2017).

The adult neuromuscular junction is an exceptionally large synapse with ca. 10^7^ nAChR molecules. Moreover, this exceptionally high number of receptors is arranged at densities of ∼10,000–20,000 μm^-2^ (Albuquerque et al., 1974; Fertuck and Salpeter, 1976; Land et al., 1977). These two characteristic features make the neuromuscular synapse a unique case, in clear contrast with the distribution and density of cholinergic receptors in brain (Borroni and Barrantes, 2021). Brain nicotinic receptors are found in the soma of neurons as well as in pre- post- and extra-synaptic regions. Of the multiple possible subunit combinations, the α4β2 and the α7 nAChRs are the most abundant oligomeric forms. Other subunit combinations and/or stoichiometries are less represented, usually in smaller quantities, and with limited regional representation (Dani and Bertrand, 2007; Taly et al., 2009).

Membrane protein topography is indivisibly paired to the host membrane composition and lateral/transversal organization of its lipid constituents. Membrane spatial organization is in turn a key modulator of membrane protein function. The tight correspondence between structure and function was finely optimized during the evolution of receptors for chemical neurotransmitters and hormones, membrane macromolecules for which efficacious signaling is closely linked to their topography and dynamics. The nAChR is a particularly well-studied case that has therefore served as a paradigmatic example of the pLGIC superfamily. This review focuses on the mechanisms of nAChR modulation by membrane lipids, organized considering the notion that the latter operate via both indirect and direct effects on membrane-embedded proteins. The nAChR fulfills the general criteria of a typical membrane-bound protein, though its abundance, density, and distinct functional properties dictate some singular modes of crosstalk with its membrane milieu.

## 2 Mechanisms of receptor protein modulation by membrane lipids

As mentioned, membrane lipids can modulate the proteins embedded in the bilayer by two non-exclusive mechanisms: direct or indirect.

### 2.1 Indirect modulation

Indirect effects of the lipid milieu on membrane-bound proteins are those exerted by general physical properties of the bulk bilayer, such as fluidity (Cooper, 1978; Mély-Goubert and Freedman, 1980; Pottel et al., 1983; Golfetto et al., 2013; de Mendoza and Pilon, 2019), elasticity (Needham and Nunn, 1990; Zeman et al., 1990; Strey et al., 1995; Chen and Rand, 1997; Bruno et al., 2007; Yersin et al., 2007), membrane curvature (Marsh, 1996b; Lundbaek et al., 1997), hydrostatic (Behan et al., 1992; Barshtein et al., 1997) and/or lateral pressure (Marsh, 1996a; Cantor, 1998; Laan et al., 2004; Heerklotz and Tsamaloukas, 2006). These physical properties, in turn, can be influenced by external factors, including drugs (Goldstein, 1984; Lehr et al., 1989), e.g., general anesthetics (Dilger et al., 1993; Franks and Lieb, 1994; Balasubramanian et al., 2002) or insecticides that alter membrane fluidity (Wolff and Bull, 1982; Moya-Quiles et al., 1996) and/or disease conditions like cancer (Schroeder, 1978; Pavel et al., 2020).

The nAChR is also a target of many of the above general, indirect modulatory factors operating via the general physical properties of the membrane bilayer. General anesthetics (Lin et al., 1991; Raines and McClure, 1997; Salord et al., 1997; Violet et al., 1997; Flood and Role, 1998; Raines and Zachariah, 1999) and alcohols are the most widely studied drugs in this regard. Straight-chain alcohols, from ethanol to octanol, inhibit the nAChR-induced current responses in a dose-dependent manner and reduce the apparent dissociation constant of the endogenous neurotransmitter, acetylcholine, by mechanisms presumably affecting membrane fluidity (Bradley et al., 1984). Patch-clamp recordings showed that both the conductance and the single-channel mean open time are reduced upon application of benzyl alcohol to muscle-type nAChRs (Bouzat and Barrantes, 1991). Ethanol is of particular relevance, since nicotine addiction is usually associated with alcohol consumption, and both drugs target the neuronal type α4β2 nAChR (Godden and Dunwiddie, 2002).

Shelby and Veatch have analyzed the indirect influences of the membrane physical state from a thermodynamic perspective and proposed that “plasma membranes have a high compositional susceptibility, arising from their thermodynamic state in a single phase that is close to a miscibility phase transition” (Shelby and Veatch, 2023). The single phase is purported to display coexisting compositional fluctuations and long-lived structures consisting of induced domains. The compositional fluctuations would provide the compositional diversity required for the multiplicity of biological functions of the plasmalemma. The model draws from the classical fluid-mosaic model of Singer and Nicolson, who proposed in 1972 that the biological membrane is a simple 2-dimensional fluid at equilibrium in which proteins and lipids are randomly distributed over distances larger than those dictated by direct interactions (Singer and Nicolson, 1972). In such a theoretical construct the lipid bilayer has been envisaged as a homogeneous phase that provides the substratum for solvating membrane-embedded proteins. The initial conceptualization has evolved over the course of decades (see the 40-year (Nicolson, 2014) and the more recent 50-year historical perspectives (Bechinger, 2023; Nicolson and Ferreira de Mattos, 2023)). Veatch and coworkers have also discussed the bilayer thermodynamic status as a fundamental property for tuning the composition of the membrane in response to external stimuli (Veatch et al., 2023).

The lipid composition of the host plasmalemma is a key, albeit indirect determinant of the conformational transitions and other functional properties of the nAChR. Early studies pointed to the lipid requirements for maintaining the receptor protein in an activatable conformational state that could eventually become unresponsive, i.e., desensitized, in the sustained presence of the agonist, (Epstein and Racker, 1978). Phospholipids were reported to stabilize the structure of the affinity purified receptor (Chang and Bock, 1979). The essential need to include cholesterol in reconstitution experiments was also an early finding (Criado et al., 1982a). Fatty acyl chain length and headgroup of the phospholipids were found to impact on the conformational equilibria of the receptor (Criado et al., 1984). Neutral and negatively charged phospholipids were analyzed in their capacity to stabilize receptor structure (Sunshine and McNamee, 1992). This led to the identification of phosphatidic acid (PA) as an anionic phospholipid playing a major role in the modulation of the nAChR (Bhushan and Mcnamee, 1993; Baenziger et al., 2000; Poveda et al., 2002; daCosta et al., 2002; Hamouda et al., 2006b; Cheng et al., 2007; Wenz and Barrantes, 2005).

Twenty years after the inception of the Singer and Nicolson model, Simons and Ikonen postulated a major challenge to the idea that the lipid bilayer constituted a homogeneous phase (Simons and Ikonen, 1997). These authors proposed the existence of lateral discontinuities in the membrane bilayer, thus introducing the concept of discrete domains whose composition differed from the rest of the membrane. They coined the term “raft” for such domains, enriched in cholesterol and sphingolipids, and drew important inferences about their functional implications, proposing that lipid rafts served as platforms for the attachment and sorting of proteins and were involved in signal transduction. Subsequent work from Simons’ laboratory explored the dynamics of glycosylphosphatidylinositol (GPI)-anchored and transmembrane protein-containing domains using single particle tracking, demonstrating the minute-long stability of discrete domains 26 ± 13 nm in diameter (Pralle et al., 2000). The degree of clustering and the dimensions of the GPI-anchored protein nanoclusters have been reported to be regulated by the lipid content -cholesterol in particular of these domains (Sharma et al., 2004).

Raft domains have been postulated to exhibit some of the characteristics of liquid-ordered (Lo) gels produced by synthetic lipid systems (Samsonov et al., 2001). Lo domains in biological membranes are characterized by their relative enrichment in cholesterol, sphingolipids and glycerophospholipids with saturated acyl chains (relative to the rest of the bilayer lipid) (see review in (Pike, 2006)). The initial characterization of “raft” domains, and the techniques employed to operationally define them, were of a biochemical nature. Homogenization of the tissue, ultracentrifugation and non-ionic detergent extraction led to the categorization of a detergent-insoluble fraction. This fraction was purported to be equivalent to the Lo-like domains in synthetic lipid systems and to occur in biological membranes.

In a recent review (Barrantes, 2023), I have critically analyzed the flaws of these biochemically-based criteria as applied to the nAChR-rich membrane domains. Using lipid mixtures with the composition of a Lo phase, no preferensolubility criteria, too broad to adequately assess the partitioning of the receptor into different lipid phases (Lichtenberg et al., 2005; Barrantes, 2007) or to be extrapolated to the conditions present in the living cell. Other direct high resolution co-localization studies are more suitable to resolve this important issue.

Indeed, Förster’s resonance energy transfer (FRET) microscopy was instrumental in providing one of the most solid experimental pieces of evidence of the nano-scale character of lipid domain structures, their active maintenance by the cell (Rao and Mayor, 2005), and their highly dynamic nature (Mayor and Rao, 2004; Sezgin et al., 2017). The “membrane domain” concept has been further expanded by experimental approaches demonstrating the effect of the underlying actin cytoskeleton on the compartmentalization and stability of lipid domains, and the various modalities (passive, neutral, active) under which the actin meshwork interacts with the membrane (Goswami et al., 2008; Raghupathy et al., 2015; Köster et al., 2016; Köster and Mayor, 2016). In its current, more complex version, the membrane domain concept encompasses a collection of lipid domain subtypes defined not only by the spatial compartmentalization produced by the lateral segregation of lipid species and their distinct chemical composition but more importantly by the signaling mechanisms they subserve (Anderson and Jacobson, 2002; Dietrich et al., 2002; Jacobson et al., 2019). This emphasis on the functional aspects was consolidated in the Keystone Symposium consensus definition of rafts as “small (10–200 nm), heterogeneous, highly dynamic sterol- and sphingolipid-enriched domains that compartmentalize cellular processes” (reviewed by (Pike, 2006)). Thus, despite their size and compositional heterogeneity, lipid domains can be defined by their ability to *indirectly* modulate membrane protein function, especially signaling.

In the field of the nAChR, raft-type lipid domains have been reported to be necessary for the stability of the α7-type nAChR in neurons (Brusés et al., 2001). Similarly, ordered lipid domains appear to be required for the clustering of nAChRs in muscle cells (Stetzkowski-Marden et al., 2006; Willmann et al., 2006; Zhu et al., 2006). The receptor-aggregating protein agrin triggers the initial steps leading to receptor aggregation in these discrete lateral lipid domains (Moransard et al., 2003; Mittaud et al., 2004). Purified nAChR reconstituted in lipid mixtures of varying composition, complexity, and morphology (single-to multi-layer vesicles, planar bilayers, giant unilamellar vesicles) (Popot et al., 1977; Anholt et al., 1980; Dalziel et al., 1980; Boheim et al., 1981; Wu and Raftery, 1981; Martinez et al., 2002) has proved to be an efficient experimental paradigm to test indirect membrane effects on membrane protein function. The purified nAChR did not show any preferential partitioning into Lo-type lipid mixtures (1:1:1 cholesterol:palmitoyloleoylphosphatidylcholine:sphingomyelin) (Bermudez et al., 2010). However, the asymmetry of the reconstituted lipid membrane resulting from inclusion of brain sphyngomyelins in the lipid mixture that modified the distribution of lipids across the reconstituted membrane induced favored nAChR partitioning in the Lo phase (Perillo et al., 2016).

Research on lipid domains in membranes has extended to the search for optimal lipid mixtures that fulfill two criteria: i) retain functional properties of the membrane protein upon affinity purification and reconstitution and ii) preserve the structure as close as possible to that of the native conformation. There has been a long search for the optimization of mild detergents and amphiphiles (Popot, 2010; Le Bon et al., 2021) to undertake structural cryo-electron microscopy studies on membrane proteins, inherently difficult to crystalize in 3 dimensions. Early attempts in this direction applied to the nAChR met with moderate success (Hertling-Jaweed et al., 1988; Schürholz et al., 1989). The more recent achievements of high-resolution studies of the nAChR by X-ray and cryo-electron microscopy (cryo-EM) methods are analyzed in Section 5.

### 2.2 Direct modulation

Direct modulation takes place when lipids exert their action upon binding to *sites* predominantly located on lipid-exposed domains of the protein. Direct modulation does not necessarily imply high affinity binding of specific lipids to the membrane protein (both low- and high-affinity binding modalities are found in Nature), though the degree of stereoselectivity is, in general, higher than that of lipids exerting indirect modulation of the membrane-embedded protein.

It was early realized that indirect effects could be targeted to the transmembrane domains of the nAChR protein (Fong and McNamee, 1986; White and Cohen, 1988; Blanton and Cohen, 1992; Fernandez-Ballester et al., 1994; Addona et al., 1998; Baenziger et al., 2000)). Exceptions to this rule can be found in the effect of ethanol on the homomeric neuronal-type α7 nAChR, which exhibits direct inhibition upon binding of the alcohol to its N-terminal extra-membranous domain (Yu et al., 1996). Using a physical technique (electron spin resonance (ESR) spectroscopy) we found that in native postsynaptic membranes from *Torpedo marmorata* electric tissue, a fraction of the lipids was relatively immobile (Marsh and Barrantes, 1978). Subsequent ESR and fluorescence experiments refined this picture (Marsh et al., 1981; Arias et al., 1990; Horvath et al., 1990) and provided information on the stoichiometry of cholesterol sites on the nAChR. About 15 cholesterol molecules could be accommodated around the transmembrane perimeter of the nAChR (Mantipragada et al., 2003). More recent work identified a discrete number of motifs with the expected amino acid residues in linear sequences favourable to the binding of cholesterol molecules. These linear motifs are found predominantly in crevices on the lipid-exposed surface of the nAChR transmembrane peptides (see Section 4.3 below).

The ESR experiments provided an additional -and unexpected-piece of information on the lipid-nAChR protein interactions: the proportion of immobilized lipid was higher than that calculated for a single boundary layer around the protein. In fact, immobilization extended to the totality of the interstitial lipid located between adjacent protein molecules in native receptor-rich postsynaptic membranes from *T. marmorata* (Marsh and Barrantes, 1978). Subsequent fluorescence quenching experiments defined a class of phospholipid sites readily exchangeable with the bulk lipid, in contradistinction to non-annular sites, accessible only to cholesterol and not to phospholipids (Jones and McNamee, 1988a). The interpretation of the fluorescence experiments in terms of “annular/non-annular” lipids was based on the additional intrinsic fluorescence quenching produced by dibromo-cholesterol when the receptor was reconstituted (and hence already partially quenched) in dibromo-dioleoyl-phosphatidylcholine liposomes. The additional quenching was assumed to unveil the presence of cholesterol sites inaccessible to phospholipids. This issue has been recently revisited, and the notion of annular vs. non-annular lipids in the context of membrane protein-associated lipid has been qualified as a myth (Gómez-Fernández and Goñi, 2022).

**FIGURE 1:**
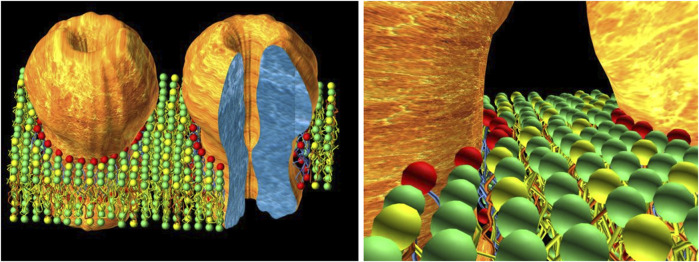
The concept of interstitial lipid. On the left, a schematic cartoon depicting the close proximity of nAChR molecules in the postsynaptic membrane, typical of the adult neuromuscular junction (and the electromotor synapse), where nAChR densities can reach values of 10,000–20,000/μm^2^ (Heuser and Salpeter, 1979). The tightly-packed 2D array of nAChRs (only two molecules are shown for the sake of clarity) leaves only a few concentric layers of lipid molecules filling the interstices between receptor macromolecules. Colored spheres represent the polar head groups of different phospholipids. The red spheres correspond to the first-layer lipid, exchanging rapidly with other layers and likely involved in direct modulation of the nAChR. The second and third layers (green and yellow spheres) beyond the first-layer lipid of a given nAChR molecule are shared without boundaries with the homologous layers of vicinal lipids surrounding adjacent neighboring receptors. The cartoon on the right shows this in more detail. This interstitial lipid thus consists of only a very few layers, with little if any “bulk” lipid in the neuromuscular junction; it is a common territory shared by neighboring receptors. The experimental basis of this concept relies on early ESR (Marsh and Barrantes, 1978; Marsh et al., 1981) and electron microscopy (Fertuck and Salpeter, 1976; Heuser and Salpeter, 1979; Salpeter and Loring, 1985). The cartoon on the right is taken from Barrantes, (2004) with permission from Elsevier.

The early ESR experimental data on native receptor-rich membranes bear direct relevance to the physical status of the lipid in the actual neuromuscular synapse. As emphasized in the Introduction, the nAChR is present at extremely high densities in quasi-crystalline 2-dimensional arrays in the neuromuscular junction (Heuser and Salpeter, 1979), as is the case with the electromotor synapse of electric fish. Hence, in these two postsynaptic membranes interstitial lipid occupies the remaining available volume between adjacent receptor macromolecules: the emerging picture is not a sea of lipids with isolated “iceberg-resembling” receptor macromolecules but rather a large 2-D “picket” of nAChRs and lipid filling in the gaps (Barrantes, 2004). This implies that each receptor cylinder, ∼8 nm in diameter, exerts a gradient of influence that extends for only a few lipid layers and is shared within a few nanometers with equivalent influences from neighboring receptors (Figure 1).

**FIGURE 2:**
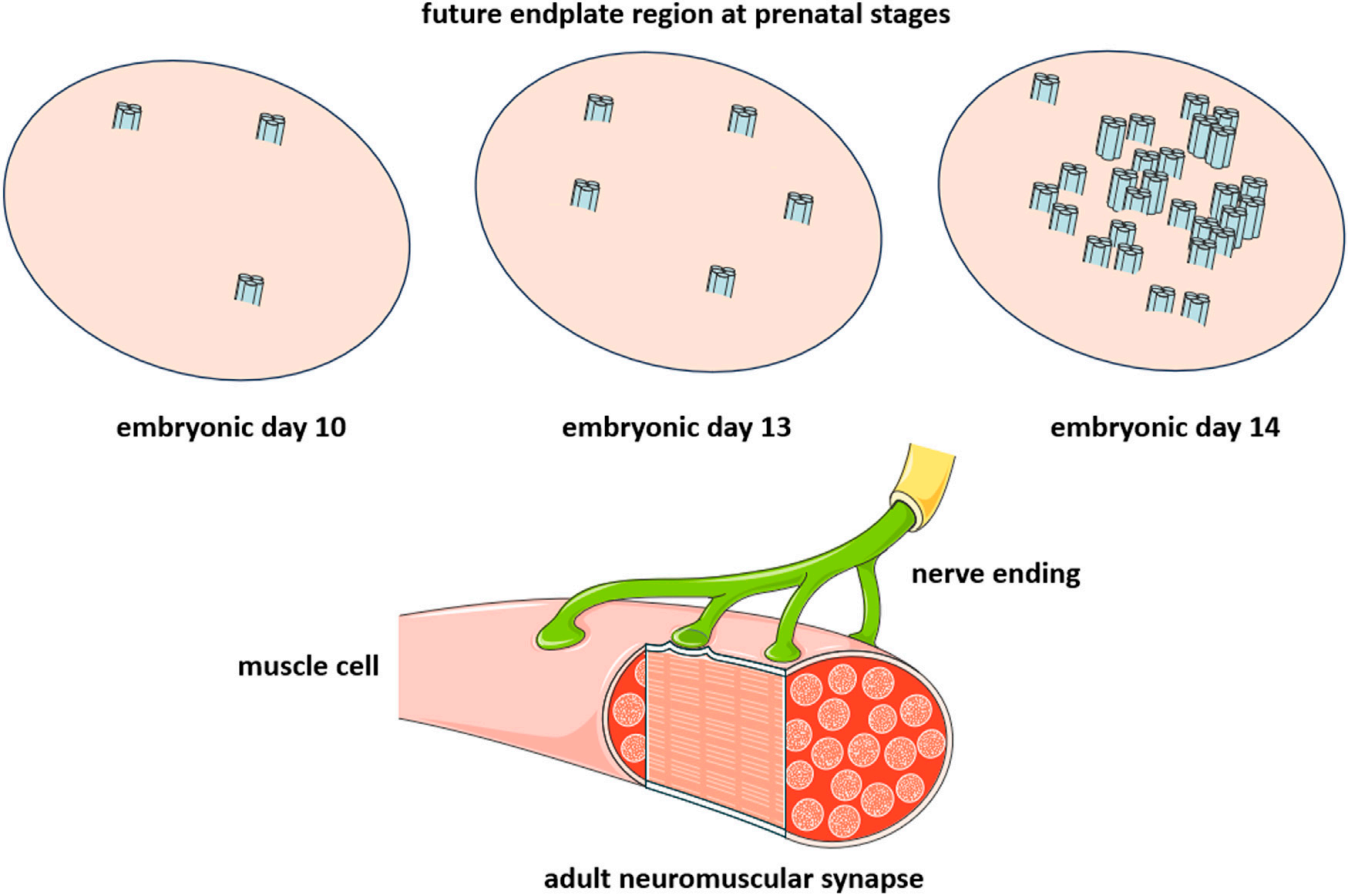
Critical developmental stage of the peripheral cholinergic synapse, the neuromuscular junction. The diagram is intended to emphasize the change occurring within a time window of 24 h between embryonic day 13 (E13) and embryonic day 14 (E14), during which individual nAChR molecules associate to form nanoclusters consisting of a few nAChR molecules. The nerve endings arrive during postnatal life (lower diagram), leading to the establishment of the neuromuscular junction (see (Sanes and Lichtman, 2001) for a review).

The lipid-protein interactions at the peripheral adult muscle-type and electromotor synapses may thus be unique and current attempts to understand them using single-molecule reconstituted systems may fall short of providing a realistic depiction of the conditions present at the uniquely large and densely crowded peripheral synapse. The valuable inferences on lipid-protein interactions drawn from the cryo-electron microscopy (see Section 5 below) and *in silico* (this Section) studies using single nAChR molecules in lipid nanodiscs are more pertinent to the conditions met at the less populated central nervous system synapses and at early ontogenetic neurodevelopmental stages of the neuromuscular junction. In the latter scenario, between embryonic day 13 and 14, individual muscle-type nAChRs diffuse in the plane of the membrane (see Section 6 on receptor dynamics below) and begin to associate (Sanes and Lichtman, 2001), in a process that I have figuratively called “receptor socialization”, leading to the formation of clusters of nanoscopic dimensions (Barrantes, 2022), different from the micron-sized clusters found in postnatal life (Figure 2). The nAChR nanoclusters likely constitute the intermediate supramolecular organization leading to the meso-scale “microaggregates” observed during the embryonic stages of neuromuscular development ((Olek et al., 1986a; Olek et al., 1986b) reviewed in (Sanes and Lichtman, 2001)) as well as in mammalian cells heterologously expressing nAChRs (Olson et al., 1983; Borroni et al., 2007).

Jonathan Cohen and Michael Blanton pioneered the use of photoaffinity labeling techniques to identify lipid sites on the nAChR molecule (Blanton and Cohen, 1992; Blanton and Cohen, 1994; Blanton et al., 2001; Hamouda et al., 2006a; Hamouda et al., 2007; Hamouda et al., 2008). Miller and coworkers employed similar approaches to characterize anesthetic sites at the lipid-nAChR interface (Forman et al., 2015). Currently there are more than 100 crystal structures of pLGICs in the Protein Data Bank (PDB). Searching for putative drug sites for general anesthetics, anxiolytics, smoking cessation drugs, and antiemetics on the transmembrane regions of the different members of the superfamily revealed the presence of recognition sites, some of which may be unique to individual receptors, while others were found to be broadly conserved among pLGIC members (Koniuszewski et al., 2022). Most recently, insecticides targeting pLGICs have been described as “structural mimetics of neurotransmitters that manipulate and deregulate” these channel proteins (Raisch and Raunser, 2023).

A minimal number of 45 lipid molecules per nAChR molecule were reported to be required to sustain ion permeation (Jones et al., 1988). Jones and McNamee found 5 to 10 “non-annular lipid” sites per nAChR monomer (Jones and McNamee, 1988b). The sterol, androstanol, was found to display a higher selectivity for the nAChR (relative to PC). Thirty-eight sterol sites per nAChR were reported by the group of McNamee (Ellena et al., 1983). ESR studies documented the presence of nAChR-immobilized PA (Ellena et al., 1983). A recent coarse-grained molecular dynamics study using a host bilayer including 30 different lipids (Sharp and Brannigan, 2021) was used to categorize lipid sites in a neuronal-type nAChR. Two classes of sites were identified: an inter-subunit site deep inside the receptor molecule, flanked by M1-M2 in a given subunit and M2-M3 of the adjacent subunit, and a peripheral, lipid-exposed site on the M4 transmembrane domain (Brannigan et al., 2008). Cholesterol was found to exhibit higher affinity for the external hemilayer. Phosphatidylinositol (PI), phosphatidylserine (PS), and phosphatidylcholines (PC) had stronger affinities for these sites than the phosphoinositides PIP1, PIP2, PIP3. The latter exhibited higher affinity than PA. From strongest to weakest, the affinities of the phospholipids for the inner hemilayer sites follow the order: PE > PI ∼ PS ∼ PC ≫ PIP1 ∼ PIP2. In the case of the M4 site, the order of affinities, from strongest to weakest, was: PE > PI > PS > PC ≫ PIP1 ∼ PIP2 ∼ PIP3 ∼ PA (Sharp and Brannigan, 2021).

The combination of biochemical and biophysical studies has contributed to establishing the important modulatory role of cholesterol in nAChR function (reviewed in (Barrantes, 1992; Barrantes, 2004). One area that still demands attention is the identification of specific sites for the sterol. An *in silico* search disclosed the occurrence of linear amino acid sequences compatible with recognition motifs CRAC and/or CARC (Baier et al., 2011). The amino acid consensus sequence termed ‘‘CRAC’’ (cholesterol recognition/interaction amino acid consensus) is usually found in the juxtamembrane region of the nAChR, with the pattern–L/V–(X) (1–5)–Y–(X) (1–5)–R/K–, where (X) (1–5) represents between one and five residues of any amino acid (Baier et al., 2011). The individual energetic contribution of the motifs was found to vary among the different transmembrane segments. The combination of mutagenesis and lipid monolayer studies established the role of key amino acid residues in determining cholesterol affinity. Recent cryo-EM studies reviewed in Section 4 below have provided experimental confirmation of these motifs in the nAChR transmembrane region (see also review in (Barrantes, 2023).

## 3 Physical state of the nAChR-vicinal lipid

Comparison of the general polarization (GP) of the fluorescent probe Laurdan and single-channel patch-clamp data obtained on two different clonal cell lines disclosed a correlation between these two parameters, pointing to the differential influence of cell-specific lipid compositions on the functional (ion permeation) properties of the receptor (Zanello et al., 1996). The inception of the FRET technique in the Laurdan GP field enabled us to examine the physical state of the interstitial lipid, i.e., the lipid microenvironment in close proximity to the receptor. The FRET pair used was the tryptophan fluorescence of the receptor (donor) and the Laurdan molecule (acceptor). Native *T. marmorata* nAChR-rich membranes and reconstituted liposomes made up of synthetic phosphatidylcholines were compared (Antollini et al., 1996). FRET-GP within donor-acceptor distances of 14 ± 1 Å served as a sensor of interstitial lipid in very close contact with the receptor protein. In coincidence with the relative lipid immobilization reported in ESR experiments (Marsh and Barrantes, 1978), the FRET-GP experiments revealed a lower polarity and an increased solvent dipolar relaxation at the hydrophilic/hydrophobic interface in native membranes relative to diluted nAChR-containing reconstituted systems (Antollini et al., 1996). In model lipid membranes, incorporation of the nAChR increases the proportion of non-hydrogen bonded lipid ester carbonyl groups (daCosta et al., 2002).

Pyrene, an organic planar molecule characterized by its extremely long fluorescence lifetime, has been used to covalently tag lipids, resulting in fluorescent probes that incorporate readily into membranes because of the highly hydrophobic fluorophore moiety. These probes form excimers When a pyrene molecule in the excited state collides with another pyrene molecule in the ground state, an excimer is formed. Successful collisions depend on both concentration and lateral diffusion of the probe (Galla and Hartmann, 1980; Holopainen et al., 1997; Somerharju, 2002). Pyrene phospholipid derivatives partition preferentially in lipid-fluid phases (Somerharju et al., 1985; Jones and Lentz, 1986; Koivusalo et al., 2004), and form probe-enriched domains (“patches”) laterally segregated from lipids in the gel state (Holopainen et al., 1997; Holopainen et al., 1998; Holopainen et al., 2001). Excimer/monomer ratiometric measurements revealed the appearance of distinct lateral heterogeneities produced by addition of the receptor protein to pure DPPC/DOPC liposomes or the same supplemented by saturated lipid (palmitoyl-oleoyl-PC or palmitoyl-oleoyl-PA). Complementary measurements of FRET from the protein to the probe diphenylhexatriene (DPH) further showed that the nAChR partitioned preferentially into POPC- or POPA-enriched, relatively more rigid domains (Wenz and Barrantes, 2005).

## 4 Cryo-EM structures reveal lipid sites on the nAChR surface

The successful combination of lipid-mimicking detergents and lipid mixtures known to support nAChR function led to the obtention of nanodiscs apt for high-resolution structural methods (Padilla-Morales et al., 2011; Quesada et al., 2016; Delgado-Vélez et al., 2021). The 3.94 Å resolution obtained by X-ray diffraction studies of the neuronal-type α4β2 nAChR provided one of the first glimpses at the atomic structure of a complete nAChR molecule (Morales-Perez et al., 2016). This was followed by studies of the other most abundant homomeric neuronal-type α7 nAChR (PDB: 7KOQ) (Noviello et al., 2021).

### 4.1 Phospholipid sites revealed by cryo-EM on the nAChR transmembrane surface

The atomic structure of the muscle-type nAChR and the localization of lipid sites was tackled more recently with cryo-EM techniques using the *Torpedo* receptor reconstituted in lipid nanodiscs. Densities attributable to phosphoglyceride sites could be resolved (Rahman et al., 2020; Rahman et al., 2022; Zarkadas et al., 2022). In the Zarkadas and coworkers study, (PDB: 7QL5), phospholipid sites (6–11) on the receptor transmembrane region could be identified, distinguishing whether the receptor was reconstituted in nanodiscs in the presence or absence of cholinergic ligands. PC sites were apparent at i) the inner, cytoplasmic facing hemilayer, sandwiched between the M3 helix of the principal subunit, and another charged residue (Lys or Arg) in the complementary M4 domain and ii) at the outer leaflet, in a shallow cavity flanked by M3, the M2-M3 loop, and the Cys loop from the principal subunit, and the M1 from the complementary subunit (Zarkadas et al., 2022). A detailed account of the nAChR-lipid structural data has recently been published (Barrantes, 2023).

### 4.2 Cholesteryl ester sites revealed in the neuronal nAChR transmembrane domains

The structure of another neuronal-type nAChR, the ganglionic α3β4 receptor, could be elucidated (PDB: 6PV7) (Gharpure et al., 2019) upon reconstitution into nanodiscs that included ion channel function-supporting lipids, supplemented with cholesteryl hemisuccinate, a water-soluble cholesteryl ester (Criado et al., 1982a), and PA. Cryo-EM densities interpreted as cholesteryl ester sites were identified on the α3β4 nAChR at the M4-M1 and the M4-M3 transmembrane interfaces of each subunit (Gharpure et al., 2019). Hibbs and coworkers had previously observed two “bowl-shaped” cholesterol sites per receptor subunit on the inner hemilayer transmembrane region in the α4β2 nAChR (Walsh et al., 2018). Each cholesterol molecule established contacts almost exclusively with a given subunit and with another adjacent cholesterol molecule in a pairwise fashion, a motif identified by Unwin in the *Torpedo* electromotor nAChR (Unwin, 2020). Cryo-EM of the detergent-solubilized nAChR protein from *Torpedo californica* in reconstituted soybean lipid-saposin nanodiscs at 2.7 Å resolution (PDB: 6UWZ) (Rahman et al., 2020) identified densities “consistent with a bound lipid” at the base of the *a* subunit M4 transmembrane domain. The possible role of cholesterol in receptor desensitization was addressed in a subsequent work (Zhuang et al., 2022).

### 4.3 Low- and high-affinity cholesterol sites revealed by cryo-EM on the *Torpedo* nAChR surface

**FIGURE 3:**
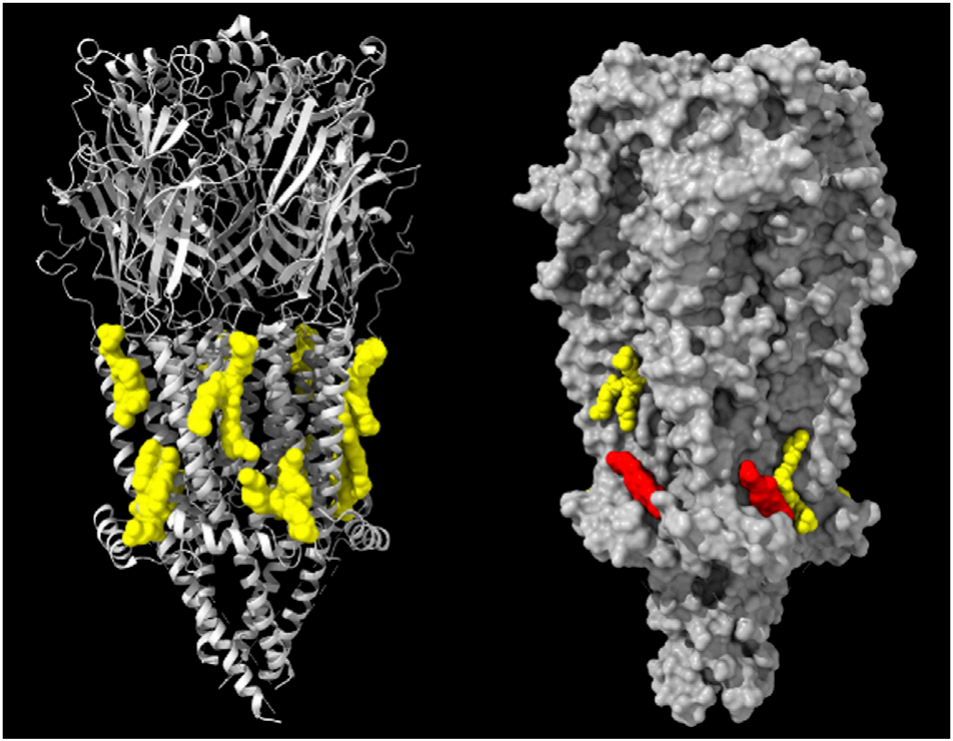
*Left:* Model derived from the cryo-EM data of the *Torpedo (Tetronarce) californica* electric organ muscle-type nAChR (grey ribbon rendering) with 8 phospholipid (phosphatidyloleoyl-PC, POPC) molecules (yellow) on both hemilayers of the receptor transmembrane region (PDB: 7QL5) (Zarkadas et al., 2022). *Right:* Model derived from the cryo-EM data of the *Torpedo californica* electric organ muscle-type nAChR (grey surface rendering) with 2 phospholipid molecules (yellow) on both hemilayers and two cholesterol molecules (red) present only in the cytoplasmic-facing hemilayer of the receptor transmembrane region (PDB: 7SMT). From ref. (Zarkadas et al., 2022). Molecular graphics performed with UCSF ChimeraX (Pettersen et al., 2004).

A cryo-EM study from Hibbs´ group reported the structure at 2.51 Å resolution of the “apo”-conformer of the *Torpedo* nAChR in the absence of ligands (PDB: 7SMM) in a soybean lipid mixture (PDB: 7SMM) (Rahman et al., 2022). Comparison of the apo-conformer with the structure of the receptor in the same lipid mixture but with added cholesterol (PDB: 7SMQ), permitted Hibbs and coworkers to distinguish cholesterol sites from phospholipid sites, and identify cholesterol sites of high- and low-affinity on the nAChR surface at the outer and inner lipid hemilayers, respectively (Figure 3). In the apo-nAChR without exogenously added cholesterol, 4 to 5 cholesterol molecules remained bound to the nAChR upon elution from affinity chromatography, and the corresponding densities identified in the inner, cytoplasmic-facing hemilayer were therefore assumed to correspond to tightly-bound endogenous cholesterols (3 per nAChR monomer). These high affinity sites are hydrophobic pockets formed by the M4, M3 and MX transmembrane domains on the principal face of the two *a* subunits and the single *ß* subunit. About 25 bound cholesterol molecules were calculated to be present in the nanodisc in samples with exogenously added cholesterol, depicting cholesterol densities at the extracellular-facing hemilayer. These outer leaflet-facing cholesterol molecules were interpreted as low-affinity cholesterol binding sites.

**FIGURE 4:**
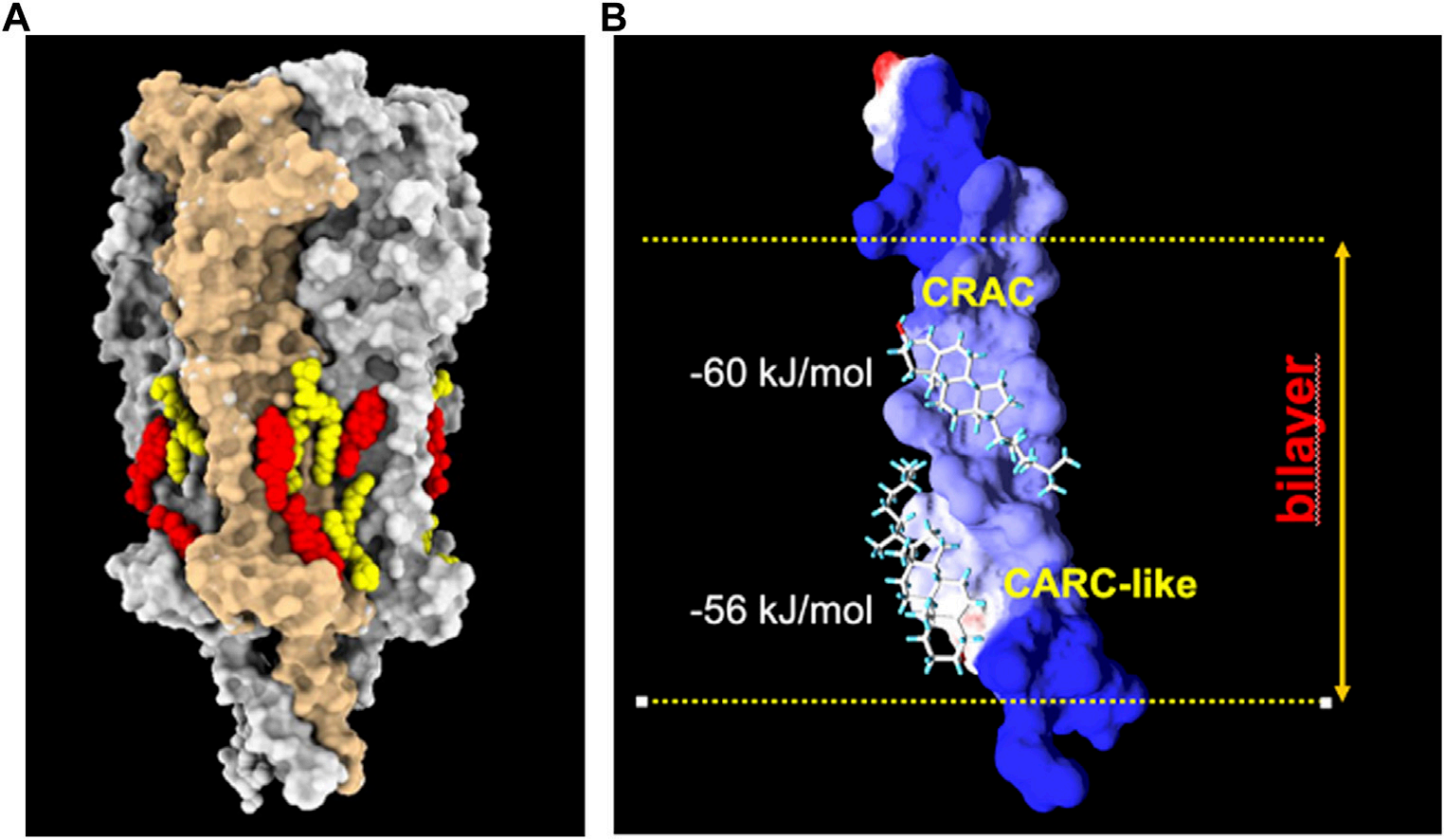
**(A)** *:* Model derived from the cryo-EM data of the *Torpedo californica* electric organ muscle-type nAChR (grey surface rendering) reconstituted in nanodisc with exogenously added soybean lipids. Under these experimental conditions, phospholipid (yellow) and cholesterol (red) molecules are observed on both hemilayers of the receptor transmembrane region (PDB: 7SMQ). Notice the preference of lipids for cavities or crevices between adjacent subunits, and the end-to-end cholesterol doublets on the inner and outer segments of the same transmembrane helices. From ref. (Rahman et al., 2022). Molecular graphics performed with UCSF ChimeraX (Pettersen et al., 2004). **(B)** *:* Cholesterol doublets in the γM4 transmembrane domain of the human nAChR, with the calculated free energy of interaction with the CRAC and CARC-like linear cholesterol consensus domains at the exofacial (top)- and cytoplasmic-facing hemilayers, respectively (reviewed in ref (Fantini et al., 2019).). Reproduced from Barrantes, 2023, an open access article distributed under the terms of the Common Access CC BY licence.

An interesting additional feature emerged from the cryo-EM atomic structures: the presence of cholesterol “doublets” on the same transmembrane segment of the nAChR (Rahman et al., 2022). This observation provided experimental confirmation of the “mirror code” motif disclosed by molecular dynamics calculations for the nAChR and a great variety of membrane-embedded proteins, including pLGICs and G-protein coupled receptors (Fantini et al., 2016). The mirror code consists of two end-to-end or tail-to-tail cholesterol recognition linear consensus domains (Figure 4). The Supplementary Information video provides a complementary dynamic 3D depiction of the *Torpedo* electric organ (muscle-type) nAChR (PDB 7QKO, (Zarkadas et al., 2022)) and cholesterol molecules on its surface (Figure 3).

## 5 Fluorescence microscopy to study the nanoscale and mesoscale dynamics of nAChRs in the plasmalemma

Learning about the modulation of nAChR dynamics by the lipid environment is particularly important for understanding the neurodevelopmental aspect of synaptogenesis. As discussed under Section 3.2 above, the motions of the receptor at embryonic stages play a fundamental role in redistributing the molecules at the muscle sarcolemma prior to the arrival of the peripheral motor nerve terminal. The nanoclusters formed at early embryonic stages precede the arrival of the nerve endings (which occurs in postnatal life). The process of receptor nanocluster assembly is fundamentally driven by lateral diffusion of individual molecules on the muscle cell membrane, thus leading to encounters with other receptor macromolecules and eventually to their aggregation. Nanoaggregates coalesce into patches of micrometric dimensions, still prior to the arrival of the nerve endings. The embryonic stages find the striated muscle cells covered with large patches (20–60 μm) that precede the fully developed muscle endplate. Translational diffusion, i.e., lateral motion, is therefore a major motor in the ontogenetic development of the neuromuscular junction, as is the case with the lateral motion of receptors from non-synaptic areas to the synaptic region in brain synapses.

Daniel Axelrod (Axelrod et al., 1976) pioneered the use of the fluorescence recovery after photobleaching (FRAP) technique for the study of nAChR translational dynamics. In postnatal developing myoblasts, the majority of the nAChRs in 20–60 μm patches are immobile; their average lateral diffusion coefficient (*D*) is <10^−4^ μm^2^ s^-1^. nAChRs in non-patched regions are diffusely distributed on the plasma membrane and display D values ∼0.5 × 10^−2^ μm^2^ s^-1^. FRAP was also applied to learn about lateral diffusion of purified nAChR monomers and dimers reconstituted into different lipid systems (Criado et al., 1982b). We compared the behavior of the two oligomeric forms of the receptor in pure dimyristoyl-phosphatidylcholine (DMPC) *versus* DMPC-cholesteryl hemisuccinate mixtures or soybean phospholipids and observed, as expected, a marked drop in diffusion coefficient as the temperature was reduced from 37°C to 14°C.

Immobilization of nAChRs is also observed in CHO-K1/A5 mammalian clonal cells expressing muscle-type nAChR but devoid of non-receptor proteins that cluster receptor molecules (Roccamo et al., 1999). FRAP combined with fluorescence correlation spectroscopy (FCS) (Baier et al., 2010) showed that ca. 55% of the receptors did not exhibit lateral motion. Cholesterol depletion reduced the fraction of mobile receptors even further (from 55% to 20%) and reduced the diffusion of the mobile receptor fraction, an observation that was subsequently interpreted as resulting from an increase in the size of the nAChR nanoclusters observed with STED nanoscopy (Kellner et al., 2007). These experiments provided evidence that nAChR nanocluster formation is strongly driven by receptor-receptor interactions and is a cholesterol-dependent phenomenon.

A subsequent series of experiments employed total internal reflection fluorescence (TIRF) microscopy to study receptor diffusion in cells tagged with fluorescent α-bungarotoxin or monoclonal anti-receptor antibodies (Almarza et al., 2014). Fluorescent α-bungarotoxin revealed a wide range of receptor mobilities including a highly mobile population (16%), a freely moving Brownian component, and a main component with restricted motion, amounting to ∼50% of the total. The rest (44%) of the nAChR molecules were considered immobile The TIRF experiments confirmed the previous FRAP/FCS results (Baier et al., 2010) showing that a large proportion of the receptors at the plasma membrane are immobile, while the mobile nAChR population is quite heterogeneous, displaying a complex spectrum of diffusional modalities, modulated by cholesterol content.

More recent experiments showed that the “highly mobile” population corresponds to superdiffusing molecules, whereas the predominant restricted motion populations correspond to various subdiffusive subpopulations (Mosqueira et al., 2018). A similar study using monoclonal antibodies (Mosqueira et al., 2020) addressed the *in vitro* effect of mAb35, an antibody that mimics the effects observed *in vivo* in the rare and severe autoimmune disease, myasthenia gravis (Tzartos and Lindstrom, 1980; Tzartos et al., 1981). Upon application of mAb35 to the CHO-K1/A5 muscle-type model cells, larger nAChR nanoclusters and slower lateral diffusion were observed (Mosqueira et al., 2020), in contrast with receptors labeled with fluorescent α-bungarotoxin, which does not crosslink the nAChR (Mosqueira et al., 2018). These biophysical studies bear relevance to the pathogenesis of myasthenia gravis: circulating anti-nAChR autoantibodies in myasthenic patients crosslink nAChR molecules and trigger their internalization (Drachman et al., 1978). Moreover, with Satyajit Mayor in Bangalore, we could reproduce the increased rate of receptor internalization upon mAb35 binding to developing myoblasts and CHO-K1/A5 cells (Kumari et al., 2008).

The combined SPT-SMLM study further showed that mAb35-induced crosslinking results in increases in the percentage of immobile nAChR molecules (∼80%) (Mosqueira et al., 2020). Moreover, antibody-tagged nAChRs interrupt their motion for periods of confinement lasting for 340–440 ms (Mosqueira et al., 2020). α-bungarotoxin tagged nAChRs also make stopovers, but for much shorter periods (135–257 ms) (Mosqueira et al., 2018).

## 6 Concluding remarks

The decades-long studies on nAChR-lipid interactions have provided a great deal of information on the modulation of the protein by membrane lipids. This review has highlighted the exceptional properties of the neuromuscular synapse, both in terms of absolute numbers and density of its receptor dotation, which dictate unique modes of lipid modulation of the protein and more importantly, underscore the significant influence of the nAChR on its interstitial lipid microenvironment. Contemporary atomistic structural studies are increasingly contributing to the identification and characterization of sites on the nAChR transmembrane domains that fulfill the requirements for lipid binding sites of low- and high-affinity and, importantly, to confirming many of the properties of these sites previously characterized by biochemical and biophysical approaches.
